# PEP-sNASP Peptide Alleviates LPS-Induced Acute Lung Injury Through the TLR4/TRAF6 Axis

**DOI:** 10.3389/fmed.2022.832713

**Published:** 2022-03-21

**Authors:** Yu-Chih Wu, Sung-Po Hsu, Meng-Chun Hu, Yu-Ting Lan, Edward T. H. Yeh, Feng-Ming Yang

**Affiliations:** ^1^School of Respiratory Therapy, College of Medicine, Taipei Medical University, Taipei, Taiwan; ^2^Department of Physiology, School of Medicine, College of Medicine, Taipei Medical University, Taipei, Taiwan; ^3^Graduate Institute of Medical Sciences, College of Medicine, Taipei Medical University, Taipei, Taiwan; ^4^Graduate Institute of Physiology, National Taiwan University College of Medicine, Taipei, Taiwan; ^5^Department of Internal Medicine, University of Arkansas for Medical Sciences, Little Rock, AK, United States; ^6^Winthrop P. Rockefeller Cancer Institute, University of Arkansas for Medical Sciences, Little Rock, AK, United States

**Keywords:** TLR4, TRAF6, inflammation, acute lung injury, NASP

## Abstract

Acute lung injury (ALI) is a severe inflammatory lung disease associated with macrophages. Somatic nuclear autoantigenic sperm protein (sNASP) is a negative regulator of Toll-like receptor (TLR) signaling that targets tumor necrosis factor (TNF) receptor-associated factor 6 (TRAF6) in macrophages, which is required to maintain homeostasis of the innate immune response. In the present study, we generated a cell permeable PEP-sNASP peptide using the sNASP protein N-terminal domain, and examined its potential therapeutic effect in a mouse model of ALI induced by the intranasal administration of lipopolysaccharide (LPS) and elucidated the underlying molecular mechanisms in RAW 264.7 cells. *In vivo*, PEP-sNASP peptide treatment markedly ameliorated pathological injury, reduced the wet/dry (W/D) weight ratio of the lungs and the production of proinflammatory cytokines (interleukin (IL)-1β, IL-6, and TNF-α). *In vitro*, we demonstrated that when the PEP-sNASP peptide was transduced into RAW 264.7 cells, it bound to TRAF6, which markedly decreased LPS-induced proinflammatory cytokines by inhibiting TRAF6 autoubiquitination, nuclear factor (NF)-κB activation, reactive oxygen species (ROS) and cellular nitric oxide (NO) levels. Furthermore, the PEP-sNASP peptide also inhibited NLR family pyrin domain containing 3 (NLRP3) inflammasome activation. Our results therefore suggest that the PEP-sNASP may provide a potential protein therapy against oxidative stress and pulmonary inflammation *via* selective TRAF6 signaling.

## Introduction

Acute lung injury (ALI) and its severe form of acute respiratory distress syndrome (ARDS) were first described in 1967 (1) and are both known to be common causes of respiratory failure in critically ill patients (2). Pathologically, ALI is often characterized by an uncontrolled acute pulmonary inflammatory response, decreased lung compliance, and impaired gas exchange, ultimately leading to various clinical disorders, such as pneumonia, pulmonary edema, and sepsis (3–5). Despite advances in understanding of the pathogenesis and treatment of this disease, the morbidity and mortality from ALI/ARDS remain high. It was estimated that the overall mortality rate of ALI/ARDS range 38.5~41.1%, which significantly depends on various comorbidities (5–7). A study conducted in Taiwan found that the in-hospital mortality rate increasing from 33.5 to 68.2% was correlated with patients' ages (8). Currently, the treatments of ALI/ARDS available in clinical practice are still limited, which emphasizes the urgent need for the development of novel therapeutic strategies to improve clinical outcomes.

Various inflammation are transmitted through a family of receptors of the innate immune system, known as Toll-like receptors (TLRs), which are known to play essential roles in the pathogenesis of pulmonary inflammation (9–12). TLR4 is the dominant mammalian receptor for the microbial product, lipopolysaccharide (LPS), a major biologically active component of gram-negative bacterial cell walls, and which is widely used to induce ALI models that are similar to pathological features of ALI in humans by triggering excessive inflammatory mediators (13–15). Our previous work showed that the somatic nuclear autoantigenic sperm protein (sNASP) is a negative regulator of the TLR4-mediated innate immune response (16, 17). In unstimulated cells, it binds tumor necrosis factor (TNF) receptor-associated factor 6 (TRAF6) to avoid excessive inflammation and maintain homeostasis of the innate immune response. Once TLR4 binds to its ligands, sNASP is phosphorylated and released from TRAF6, which ultimately stimulates expressions of proinflammatory cytokines, such as interleukin (IL)-6 and TNF-α. In addition, using a cecal ligation and puncture (CLP)-induced sepsis model with dominant-negative sNASP mutant mice, these mutant mice were more susceptible to bacterial infection due to ineffective clearance of bacteria in the lungs after CLP, indicating that the sNASP may be involved in pathological changes in lung inflammation. Those results suggested that sNASP functions as a negative regulator of innate immunity and may contribute to its protective effects against ALI and underling mechanisms.

PEP-1 is a 21-residue peptide carrier that can efficiently transduce proteins into various cells and tissues (18, 19). It consists of three domains: (1) a hydrophobic tryptophan-rich motif (KETWWETWWTEW) for targeting cell membranes, (2) a spacer (SQP), and (3) a hydrophilic lysine-rich domain (KKKRKV) for intracellular delivery and peptide solubility. Recently, it was shown that PEP fusion proteins successfully protected against various diseases including lung inflammation and neuronal diseases (20–22). In this study, we designed a PEP-NASP fusion protein to investigate whether the transduced PEP-NASP protects cells against LPS-induced inflammation. We found that PEP-NASP was efficiently transduced into RAW 264.7 cells through a membrane barrier, and that the transduced PEP-NASP reduced LPS-induced proinflammatory cytokines and nitric oxide (NO) production as well as NLR family pyrin domain containing 3 (NLRP3) inflammasome activation. *In vivo*, administration of the PEP-NASP markedly attenuated LPS-induced lung injury, with significant reductions in neutrophil infiltration, myeloperoxidase (MPO) activity, and bronchoalveolar lavage fluid (BALF) protein content. Therefore, we suggest that the PEP-NASP fusion protein might be useful as a potential therapeutic agent against ALI.

## Materials and Methods

### Mice and LPS-Induced ALI Model

Pathogen-free, 8-week-old male C57BL/6 mice were purchased from the National Laboratory Animal Center (Taipei, Taiwan). Mice were housed in a pathogen-free animal facility at Taipei Medical University (Taipei, Taiwan). Animal protocols described in this study were approved by the animal and ethics review committee of the Laboratory Animal Center at Taipei Medical University.

ALI was induced by intranasal (i.n.) instillation of LPS [5 mg/kg body weight (BW)] as described previously (23, 24). LPS was obtained from Sigma-Aldrich (St. Louis, MO, USA). Forty mice were randomly divided into four groups: sham+NASP, sham+PEP-NASP, LPS+NASP, and LPS+PEP-NASP groups. Mice in the sham and LPS groups received i.n. instillation of phosphate-buffered saline (PBS) and LPS (5 mg/kg), respectively, while mice in the LPS+NASP and LPS+PEP-NASP groups received an i.n. injection of NASP or PEP-NASP (0.1 mg/kg) at 1 h before LPS inhalation. The experiment was terminated at 24 h after LPS inhalation, after which lung tissues and BALF were harvested and collected.

### Preparation of the PEP-NASP

The His-PEP-NASP (HHHHHHKETWWETWWTEWSQPKKKRKVMAMESTATAAVAAELVSADKIEDVPAPSTSA) and control His-NASP (HHHHHHMAMESTATAAVAAELVSADKIEDVPAPSTSA) fusion proteins were synthesized by Biotools (Taipei, Taiwan). All peptides were dissolved in 0.1% DMSO and stored at −80°C.

### Cell Culture and Transduction of the PEP-NASP

The mouse macrophage RAW 264.7 cell line (ATCC TIB-71, American Type Culture Collection, Manassas, VA, USA) and embryonic kidney epithelial HEK293 cell line (ATCC CRL-1573) were both cultured in Dulbecco's modified Eagle medium (DMEM) supplemented with 10% fetal bovine serum (FBS), and 1% penicillin and streptomycin at 37°C and 5% CO_2_ in air as previously described (16).

To determine the dose-dependent transduction ability of the PEP-NASP, cells were grown to 75% confluency for 24 h, and then various concentrations (0.1 ~ 0.4 μM) of the PEP-NASP were added to the culture medium for 1 h at 37 °C. For time-dependent transduction, cells were treated with 0.4 μM of the PEP-NASP for 10 ~6 0 min. To determine the intracellular stability of the transduced PEP-NASP, cells were treated with 0.4 μM PEP-NASP for 1 h and washed with fresh medium to remove fusion proteins that had not been transduced into cells. Cells were further incubated for 60 h.

### Plasmids and Transfection

Plasmids encoding Flag-TRAF6 and green fluorescent protein (GFP)-sNASP were previously described (16). After the pGFP-sNASP plasmid had been digested with SbfI/ApaI or EcoNI/ApaI, the ends were blunted with Klenow and then self-ligated to generate pGFP-sNASP 1-233 (dTRP3) and pGFP-sNASP 1-348 (sNLS). The pGFP-sNASP 1-31 (dTRP1/2/3), 1-93 (dTRP2/3), and 94-449 (dTRP1) plasmids were constructed by polymerase chain reaction (PCR) amplification using relevant primers incorporating a KpnI site and an ApaI site, and the resulting fragment was inserted into the KpnI/ApaI-digested pGFP-sNASP. Transfection of HEK293 and RAW 264.7 cells was performed using Lipofectamine 3000 (L3000015, Invitrogen, USA) according to the manufacturer's protocol.

### Immunoprecipitation (IP) and Western Blotting

Harvested cells were subjected to IP, lysates were resolved on sodium dodecylsulfate polyacrylamide gel electrophoresis (SDS-PAGE), and a Western blot analysis was performed as previously described (16, 25). Signals were detected using the Trident femto Western horseradish peroxidase (HRP) substrate (GTX14698, GeneTex, Taiwan). Images were captured and the density of the bands was measured using the UVP BioSpectrum 815 (Jena, Germany). The detected bands were quantitated using ImageJ image analytical software. Antibodies used for the immunoblot analysis were mouse anti-NASP (SC-161915, Santa Cruz Biotechnology, Santa Cruz, CA, USA), mouse anti-TRAF6 (SC-7221, Santa Cruz), mouse anti-ubiquitin (SC-8017, Santa Cruz), mouse anti-β-actin (A1544, Sigma-Aldrich, St. Louis, MO, USA), mouse anti-phospho-TAK1 (4531, Cell Signaling, USA), mouse anti-TAK1 (4505, Cell Signaling), mouse anti-phospho-p38 mitogen-activated protein kinase (MAPK) (9211, Cell Signaling), anti-p38 MAPK (9212, Cell Signaling), mouse anti-phospho-c-Jun N-terminal kinase (JNK) (4671, Cell Signaling), mouse anti-JNK (9252, Cell Signaling), mouse anti-GFP (632281, Clontech, USA), mouse anti-COS-2 (SC-19999, Santa Cruz), mouse anti-His (SC-8036, Santa Cruz), mouse anti-Flag (F1804, Sigma-Aldrich), rabbit anti-inducible NO synthase (iNOS) (2982, Cell Signaling), rabbit anti-NLRP3 (15101, Cell Signaling), rabbit anti-capase-1 (GTX11701, GeneTex), and rabbit anti-ASC (67824, Cell Signaling). Antibodies were diluted according to the manufacturers' instructions.

### RNA Isolation and Real-Time Quantitative (q)PCR

RNA was extracted from cells using the Toolsmart RNA extractor (TB-DPT-BD24, Biotools), and then reverse-transcribed into complementary (c)DNA with a magic RT cDNA synthesis kit (Bio-Genesis Technologies, Taipei, Taiwan) according to the manufacturer's instructions. qPCRs were performed using TaqMan Gene Expression Master Mix (4369016, Thermo Fisher Scientific, Waltham, MA, USA) in an StepOnePlusTM machine (Thermo Fisher Scientific, Waltham, MA, USA). The TaqMan Gene Expression Assay used as follows: *IL-6* (Mm00446190_m1), *TNF-*α (Mm00443260_g1), and *IL-1*β (Mm00434228_m1, was all from Thermo Fisher Scientific, Waltham, MA, USA). Results were normalized to the expression of the gene encoding 18s and were quantified by the change-in-threshold method (ΔΔCT).

### Enzyme-Linked Immunosorbent Assay (ELISA)

Concentrations of cytokines in bronchoalveolar lavage fluid (BALF) and cell culture supernatants were measured using Mouse Ready-Set-Go ELISA for IL-6 (88-7064), TNF-α (88-7324), and IL-1β (88-7013) purchased from BioLegend (San Diego, CA, USA), according to the manufacturer's protocol.

### Luciferase Assays

Luciferase reporter assays were conducted according to our previous publication (26). RAW 264.7 cells were subcultured in 24-well tissue culture plates and transfected with NF-κB luciferase and pRL-TK Renilla luciferase. After 24 h, cells were transduced with the NASP or PEP-NASP 1 h before LPS stimulation, and then cells were lysed with lysis buffer, and firefly luciferase, and Renilla luciferase activities were determined using the Dual-Glo Luciferase Assay System (E2920, Promega, Madison, WI, USA).

### Immunofluorescence (IF) Staining

IF staining was conducted as described previously (27). Cells were maintained on 12-mm glass slides for 24 h and then were stimulated with 1 μg/mL LPS for 30 min in the presence of the NASP or PEP-NASP (0.4 μM) added 1 h before stimulation. Then cells were fixed with 4% paraformaldehyde for 10 min and blocked with Blocking One Histo (06349-64, Nacalai Tesque, Kyoto, Japan) for 1 h, followed by incubation with rabbit anti-NF-κB p65 Alexa Fluor 594 (SC-8008 AF594, Santa Cruz) and mouse anti-His antibodies (34650, Qiagen, USA) overnight at 4 °C. After being washed, cells were incubated with an FITC-labeled goat anti-mouse immunoglobulin G (IgG) antibody for 1 h. Nuclear DNA was stained with DAPI for 10 min. Images were captured using confocal microscopy on a Leica TCS SP5 (Wetzlar, Germany).

### Histological Analysis

Hematoxylin and eosin (H&E) staining of paraffin-embedded lung tissue sections was performed to determine cellular infiltration. Lung tissues were fixed with 10% buffered formalin for 24 h, embedded in paraffin, and sectioned at 4-μm thickness. After deparaffinization and dehydration, sections were stained with H&E and were observed with an Echo Revolve Fluorescence Microscope (San Diego, CA, USA).

### Lung Wet/Dry (W/D) Ratio

The left lung was harvested and weighed to obtain the wet weight. The lung was then placed into a drying oven at 80°C for 48 h to obtain the dry weight. The W/D weight ratio was calculated to evaluate the degree of lung edema.

### Myeloperoxidase (MPO) Activity Assay

Lung tissues were homogenized with MPO assay buffer after collection and then centrifuged at 13,000 g for 10 min at 4°C. The supernatant was assayed for MPO activity according to the manufacturer's instructions (K744-100, Biovision, USA). MPO activity was One unit of MPO activity was defined as the quantity of enzyme that converted 1 μmol hydrogen peroxide to water each minute at 37°C. MPO activity is expressed as units per gram (U/g) wet weight.

### Bronchoalveolar Lavage Fluid (BALF) Collection and Analysis

BALF was harvested according to a previous description (28). Briefly, mice were euthanized, and a catheter was installed in the open trachea. Then, 0.8 ml of ice-cold PBS was instilled into the lungs three times, and the recovered BALF was centrifuged at 450 ×*g* for 10 min. The cell pellet was used to count numbers of total cells, macrophages, and neutrophils counted with a hemocytometer and Wright-Giemsa staining (K1438-30, Biovision, USA).

### Measurement of Reactive Oxygen Species (ROS) and Glutathione (GSH) Level

ROS and GSH level were estimated by using Fluorometric Intracellular ROS Kit (MAK144, Sigma-Aldrich, St. Louis, MO, USA) and Reduced Glutathione (GSH) Assay Kit (MAK364, Sigma-Aldrich, St. Louis, MO, USA), respectively, according to the manufacturer's instructions. Fluorescence intensity was measured using a SpectraMax^®^ i3x microplate reader (Molecular Devices, San Jose, CA, USA).

### Cell Viability Determination

The Cell Proliferation Kit I (MTT) (11465007001, Merck, USA) was used to detect cell viability. According to the manufacturer's instructions, cells were cultured onto a 96-well plate under the indicated conditions. At the indicated time, 10 μl of MTT labeling reagent were added into each well and incubated at 37°C for 4 h. Then 100 μl of Solubilization solution was added to dissolve the purple formazan crystals. Absorbance at wavelength 570 nm was detected with a SpectraMax^®^ i3x microplate reader (Molecular Devices, San Jose, CA, USA).

### Statistical Analysis

GraphPad Prism 6 software was used for all statistical analyses by Student's *t*-test for two groups or a one-way analysis of variance (ANOVA) for three or more groups. Values of *p* < 0.05 were considered statistically significant.

## Results

### N-Terminal Region of sNASP Physically Interacts With TRAF6

**Figure 1:**
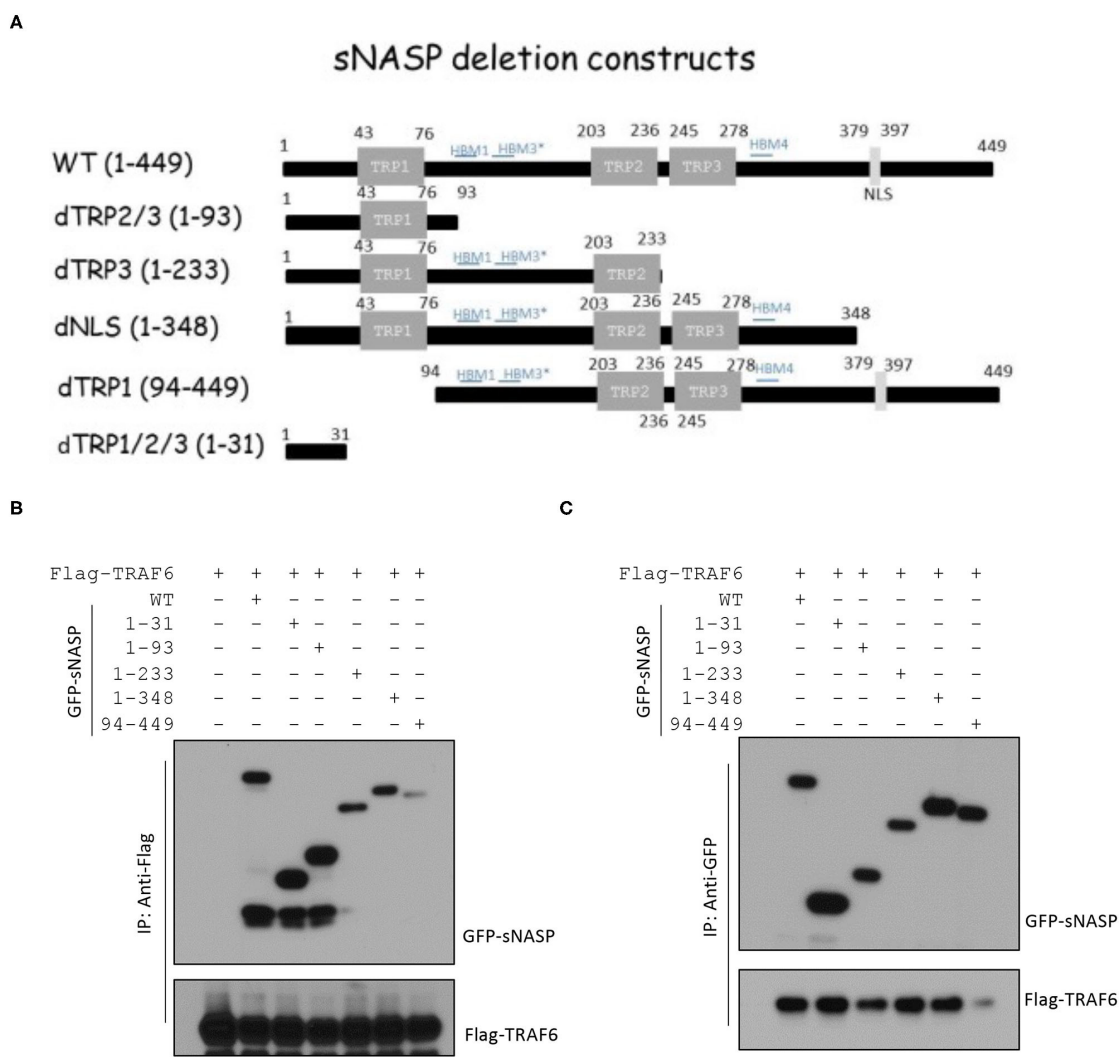
The N-terminal 1~31 domain of the somatic nuclear autoantigenic sperm protein (sNASP) is essential for interaction with tumor necrosis factor receptor-associated factor 6 (TRAF6). **(A)** Schematic representation of sNASP deletion constructs fused to the C-terminal enhanced green fluorescent protein (EGFP) tag. **(B,C)** Immunoprecipitation (IP) of Flag-TRAF6 **(B)** (with anti-Flag agarose) or GFP-sNASP **(C)** (with GFP-Trap) from HEK293 cells transiently transfected with GFP-sNASP wild-type or deletion mutants plus Flag-TRAF6, followed by immunoblotting (IB) with an antibody against Flag or GFP. Data represent a minimum of three independent experiments.

In our previous publication, sNASP was identified as a TRAF6-binding protein (16). The sNASP protein has three tetratricopeptide repeat (TPR) motifs (amino acids 43 ~ 76, 203 ~ 236, and 245 ~ 278), a region of coiled-coil domains (CCDs), and a nuclear localization sequence (NLS; amino acids 379 ~ 397) (29). To identify specific regions of the sNASP that interacts with TRAF6, various sNASP deletion mutants were generated with GFP-tagged fusion proteins (Figure 1A). Reciprocal IP and Western blot analyses confirmed that sNASP binds to TRAF6 (Figures 1B,C and Supplementary Figure 1). The N-terminal region (amino acids 1 ~ 31) of sNASP, which contains the ring and zinc fingers, was able to bind to TRAF6. Deletion of the N-terminal sNASP abolished the association with TRAF6 (Figures 1B,C and Supplementary Figure 1). Among sNASP deletion mutants, TRAF6 bound to the dTRP1/2/3 (amino acids 1 ~ 31), dTRP2/3 (amino acids 1 ~ 93), dTRP3 (amino acids 1 ~ 233), and dNLS (amino acids 1 ~ 348) mutants but not to dTRP1 (amino acids 94 ~ 449), indicating that the minimal N-terminal of sNASP containing residues 1 ~ 31 is required for TRAF6 interactions.

### Transduction of the PEP-NASP Into RAW 264.7 Cells

TRAF6 is a crucial mediator that transmits intracellular signals through TLRs to a wide range of immunostimulatory cytokines and chemokines against invading microorganisms (9, 30, 31). In this study, sNASP residues 1 ~ 31 fused with PEP-1 (PEP-NASP) were synthesized to determine whether the increase in sNASP by protein transduction had a negative regulatory effect on the LPS-induced inflammatory response in the mouse macrophage RAW 264.7 cell line.

**Figure 2:**
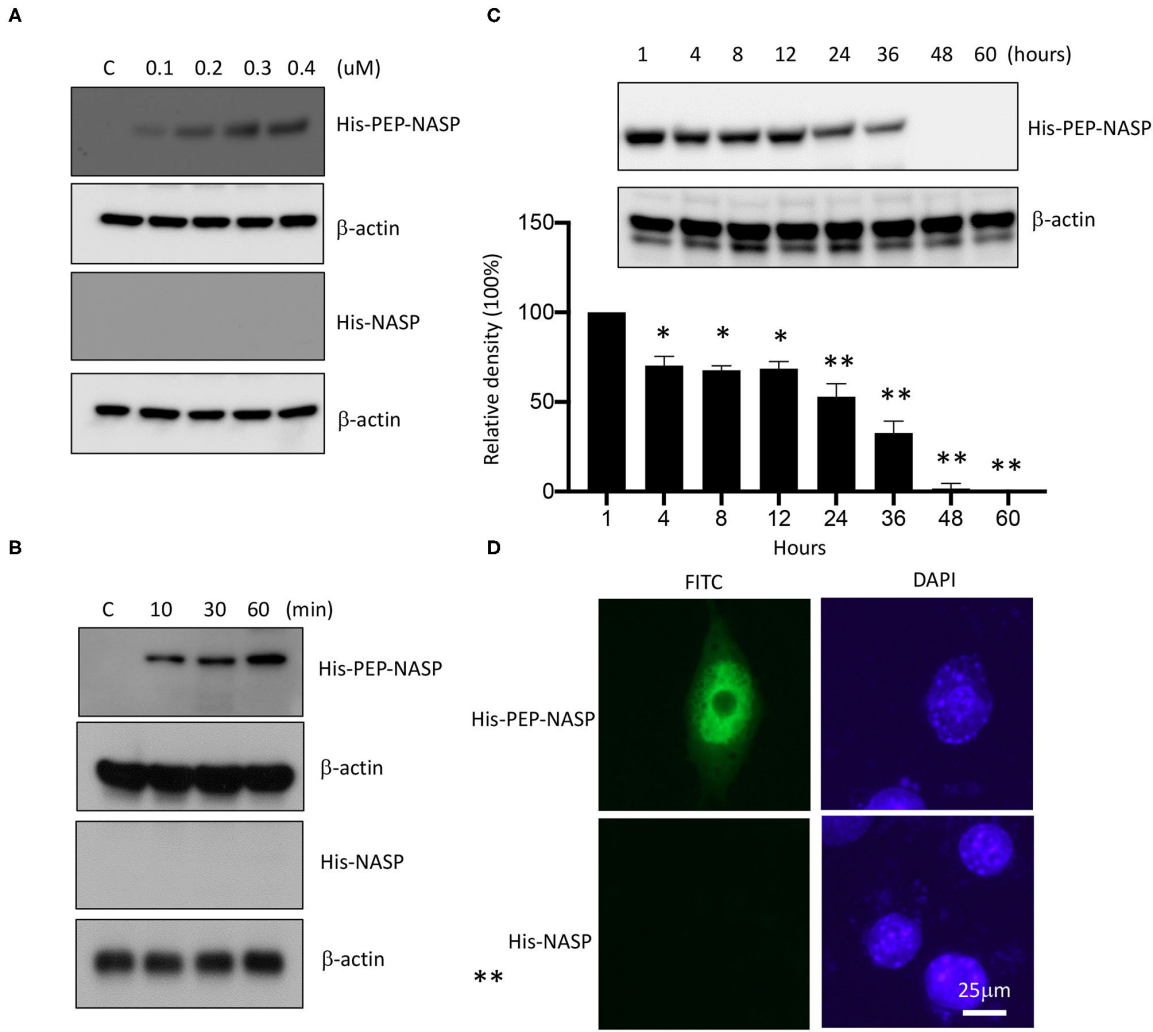
Transduction of the PEP-NASP into RAW 264.7 cells. Cells were cultured in 60-mm culture dishes. Then, PEP-NASP (0.1 ~ 0.4 μM) and control NASP were added to the culture media for 1 h **(A)**, and PEP-NASP (0.4 μM) and control NASP were added to the culture media for 10 ~ 60 min **(B)**, and analyzed by immunoblotting (IB) with an antibody against His or β-actin. Transduction of PEP-NASP stability was assessed after various time periods (1 ~ 60 h). Cells pretreated with 0.4 μM PEP-NASP for 1 h and analyzed by IB with an antibody against His or β-actin **(C)**. The intracellular distribution of the transduced PEP-NASP and NASP was observed by confocal microscopy **(D)**. Scale bar = 25 μm. Data represent a minimum of three independent experiments.

To evaluate the transduction ability of the PEP-NASP, RAW 264.7 cells were incubated with various concentrations (0.1 ~ 0.4 μM, 1 h) of the PEP-NASP and for various times (10 ~ 60 min, 0.4 μM). Then protein transduction was analyzed by Western blotting with an anti-His antibody. As shown in Figures 2A,B, the PEP-NASP was successfully transduced into RAW 264.7 cells in dose- and time-dependent manners, whereas the control NASP lacking the PEP-1 domain was not transduced into cells. The intracellular concentration of the transduced PEP-NASP in cells was detected within 10 min and had gradually increased by 60 min (Figure 2B). Since stability is an important factor in protein therapy, we examined the stability of the transduced PEP-NASP at various time periods. Western blotting revealed that the transduced PEP-NASP in RAW 264.7 remained stable until 36 h after treatment, subsequently decomposing over time (Figure 2C). The viability of RAW 264.7 cells wasn't affected when treated with PEP-NASP, suggesting that PEP-NASP did not cause cytotoxicity in cells (Supplementary Figure 2). In addition, cellular localization of the transduced PEP-NASP in cells was determined by IF staining. PEP-1-NASP fluorescence was distributed in both the nucleus and cytoplasm, but no fluorescent signal was observed in control NASP-transduced cells (Figure 2D). These results demonstrated that the PEP-NASP was efficiently transduced into cells and persisted for 36 h after transduction.

### Transduced PEP-NASP Inhibits the LPS-Induced Inflammatory Response Through TRAF6

**Figure 3:**
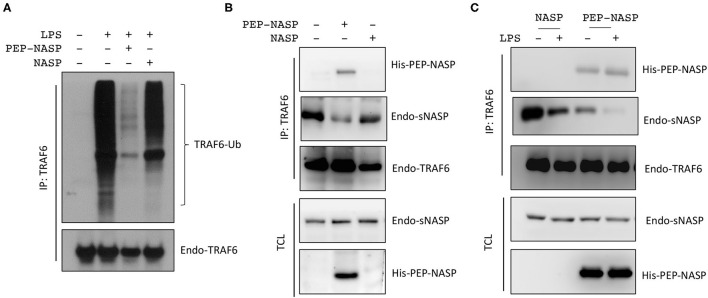
The PEP-NASP significantly inhibits tumor necrosis factor receptor-associated factor 6 (TRAF6) polyubiquitination in LPS-stimulated RAW 264.7 cells. **(A)** Cells were pretreated with or without the PEP-NASP or NASP (0.4 μM) for 1 h before the addition of LPS (1 μg/mL) for 30 min. Cell lysates were immunoprecipitated with an anti-TRAF6 followed by immunoblotting (IB) with an antibody against Ub or TRAF6. **(B)** Immunoprecipitation (IP) of endogenous TRAF6 (with anti-TRAF6) from RAW 264.7 cells transduced with the PEP-NASP or NASP, followed by IB with an antibody against His, NASP, or TRAF6. Total cell lysate (TCL) IB was done with anti-NASP and anti-His. **(C)** Cells were transduced with the PEP-NASP or NASP, stimulated with LPS, and assessed by IB with an antibody against His, NASP, or TRAF6 after IP with anti-TRAF6 or by IB with anti-NASP and anti-His in TCL. Data represent a minimum of three independent experiments.

Our previous report showed that sNASP binds to TRAF6 and inhibits TRAF6 autoubiquitination and its downstream signaling (16); we wanted to evaluate whether the transduced PEP-NASP would affect TRAF6 autoubiquitination following LPS stimulation. Results indicated that transduction of the PEP-NASP, but not the control NASP, diminished LPS-induced TRAF6 autoubiquitination in RAW 264.7 cells (Figure 3A). Next, we investigated whether the PEP-NASP could interact with endogenous TRAF6 during regulation of TLR4 signaling. There was a clear interaction between endogenous TRAF6 and the PEP-NASP, but not the control NASP (Figures 3B,C). IP assays also showed that transduction of the PEP-NASP led to reduced interactions between endogenous sNASP and TRAF6 (Figure 3B). Following LPS stimulation, transduction of the PEP-NASP accelerated the dissociation of the sNASP from TRAF6 (Figure 3C), indicating that the PEP-NASP had a much higher affinity for endogenous TRAF6 than endogenous sNASP. These results suggested that transduction of the PEP-NASP may compete with endogenous sNASP for the TRAF6-binding motif.

**Figure 4:**
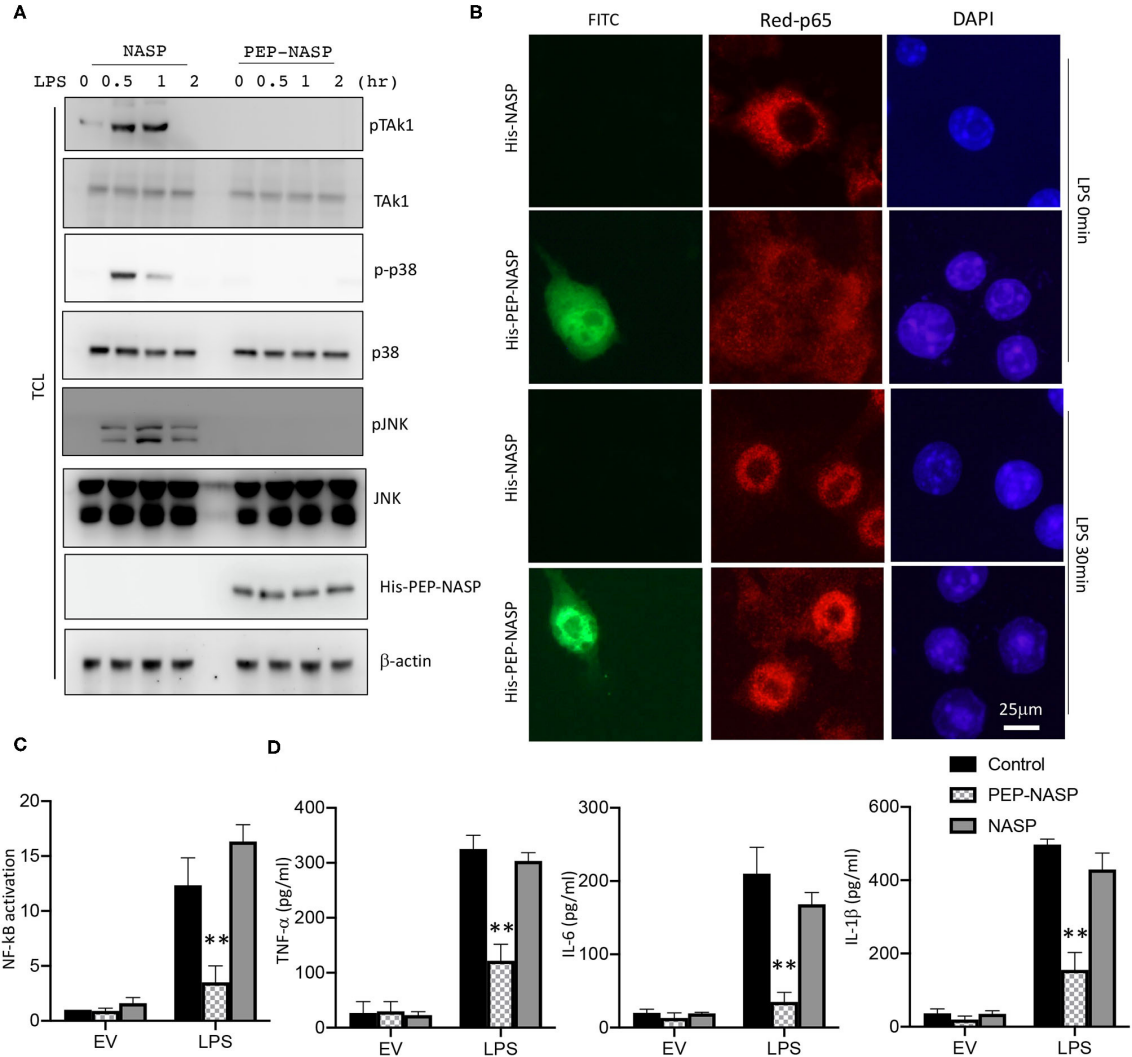
The PEP-NASP inhibited LPS-induced activation of NF-κB and expression of proinflammatory cytokine production in macrophages. **(A)** RAW 264.7 cells pretreated with the PEP-NASP or NASP were stimulated with LPS for different time points and assessed by an immunoblotting (IB) analysis with the indicated antibodies. **(B)** FITC-labeled PEP-NASP or NASP was transduced into RAW 264.7 cells. After 48 h, cells were fixed and immunostained with an anti-p65 (Red) antibody. Nuclei were counterstained with DAPI. Images were visualized by confocal microscopy. Scale bar = 25 μm. **(C)** Luciferase activity in RAW 264.7 cells transfected with a luciferase reporter vector driven by an NF-κB-responsive promoter, plus the control, PEP-NASP, or NASP. Results were standardized to an empty vector (EV; set to 1). Data are the mean ± standard error (SE) for each group. **(D)** Production of TNF-α, IL-6, and IL-1β by RAW 264.7 cells transduced with the PEP-NASP or NASP and stimulated with LPS. Data are the mean ± SE for each group. **p < 0.01 (by a one-way ANOVA). Data represent a minimum of three independent experiments.

Since NF-κB activation is crucial for the LPS/TLR4 signaling-induced inflammatory response in RAW 264.7 cells, we examined the effect of the PEP-NASP against LPS-induced TRAF6 downstream signaling and the nuclear translocation of p65 in RAW 264.7 cells following LPS stimulation. As shown in Figure 4A, LPS-induced phosphorylation of transforming growth factor (TGF)-β activated kinase 1 (TAK1), p38 MAPK, and JNK decreased when the PEP-NASP was transduced into RAW 264.7 cells. Furthermore, the amount of NF-κB p65 in the nucleus markedly increased upon exposure to LPS alone, but the PEP-NASP suppressed the LPS-mediated nuclear translocation of NF-κB p65 (Figure 4B). Transduced PEP-NASP, but not control NASP, was also found to inhibit LPS-mediated NF-κB activation (Figure 4C). In additional, the PEP-NASP significantly decreased the production of the proinflammatory cytokines: TNF-α, IL-6, and IL-1β (Figure 4D). These results suggested that the transduced PEP-NASP negatively regulates activation of the LPS-triggered TLR4 pathway by regulating TRAF6 autoubiquitination.

### PEP-NASP Suppresses LPS-Induced ROS/NO Production and NLRP3 Inflammasome Activation

**Figure 5:**
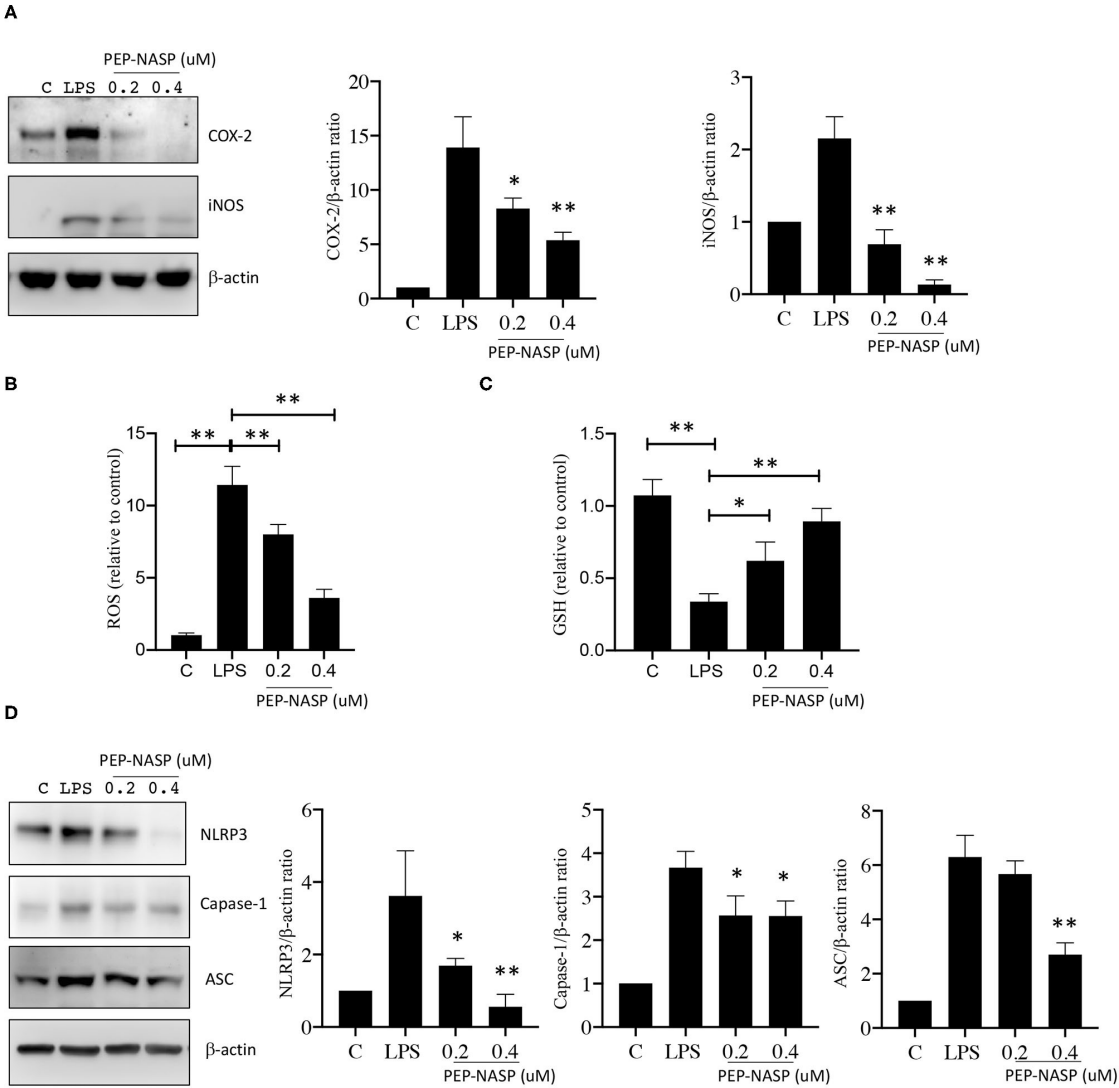
LPS-induced NLRP3 inflammasome and NO production were inhibited by the PEP-NASP. **(A)** Protein expression levels of COX-2, iNOS, and β-actin in RAW 264.7 cells pretreated with the PEP-NASP and stimulated with LPS as detected by a Western blot analysis. **(B)** Production of ROS, **(C)** GSH level and **(D)** Protein expression levels of NLRP3, caspase-1, ASC, and β-actin in RAW 264.7 cells transduced as in A and stimulated with LPS. Western blots were quantified by normalizing densitometric values to those of β-actin. Data are expressed as mean ± standard error for each group. Signal values were compared to unstimulated conditions. *p < 0.05, **p < 0.01 (by a one-way ANOVA). Data represent at least three independent experiments.

The production of excessive ROS and NO after LPS challenge is an important reflection of the inflammatory process involved in local immune responses. Since iNOS and cyclooxygenase (COX)-2 proteins are associated with NO production, we assessed whether the LPS-induced production of NO mediated by iNOS and COX-2 was regulated by the transduced PEP-NASP. As shown in Figure 5A, expressions of the iNOS and COX-2 proteins significantly increased in LPS-stimulated RAW 264.7 cells compared to unstimulated cells. However, the transduced PEP-NASP dose-dependently (0.2 and 0.4 μM) inhibited expressions of iNOS and COX-2 in the presence of LPS (Figure 5A). Similar result was found in ROS production. As shown in Figures 5B,C, ROS production significantly decreased, and GSH, as the major free radical scavenger, level significantly increased in PEP-NASP transduced group compared with the LPS group. These results implied that the PEP-NASP inhibited ROS and NO production by downregulating iNOS and COX-2 expressions.

Our study showed that the PEP-NASP could reduce IL-1β production in LPS-induced RAW 264.7 cells (Figure 4D), and the NLRP3 inflammasome was reported to be essential to turn the inactive precursor (pro-IL-1β) into the active form (IL-1β). Consequently, to elucidate whether its anti-inflammatory effect was related to the NLRP3 inflammasome, we further explored the effect of the PEP-NASP on activating the NLRP3 inflammasome. As shown in Figure 5D, LPS challenge significantly enhanced expressions of NLRP3, ASC, and caspase-1 proteins, indicating that the NLRP3 inflammasome was activated by LPS in macrophages. PEP-NASP transduction dramatically blocked the LPS-induced upregulation of NLRP3, ASC, and caspase-1 compared to treatment with LPS alone (Figure 5D). These results indicated that the PEP-NASP might reduce promotion of the NLRP3 inflammasome to inhibit inflammation.

### PEP-NASP Transduction Attenuates LPS-Induced Lung Injury in Mice

**Figure 6:**
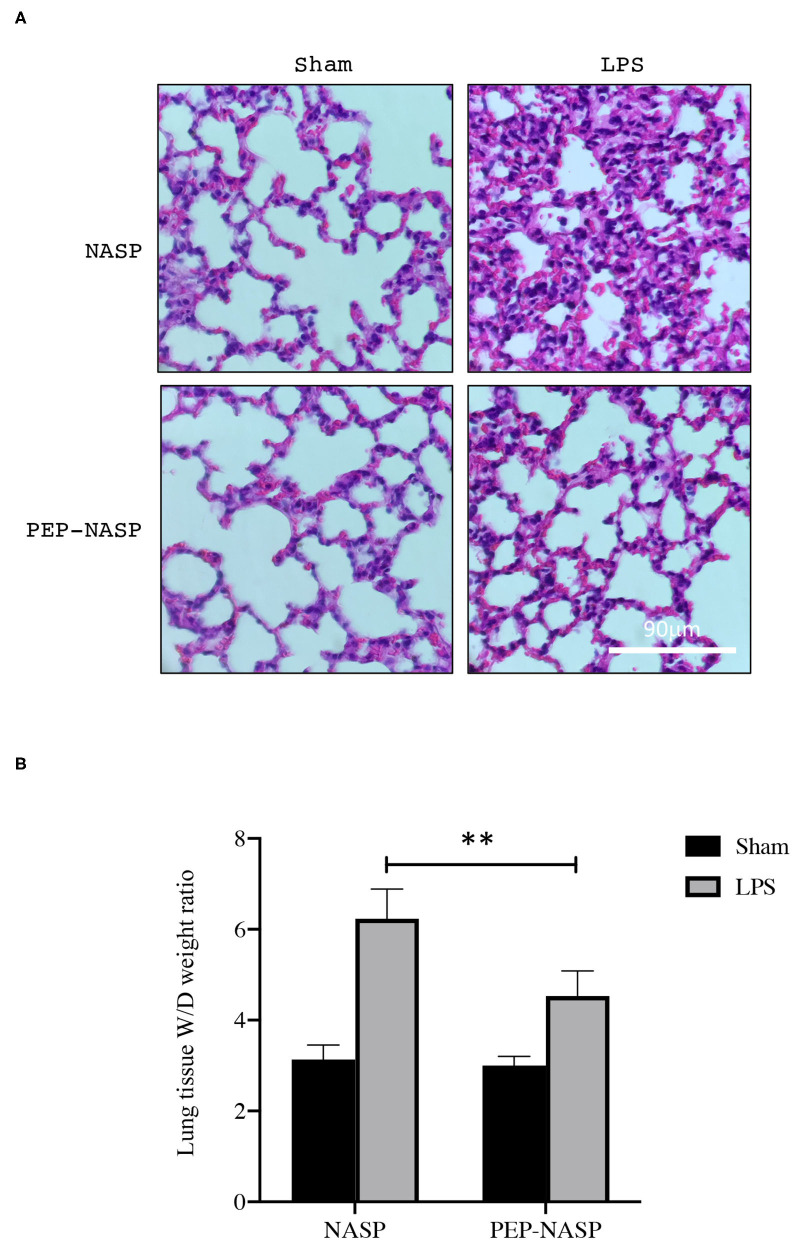
The PEP-NASP significantly suppresses LPS-induced acute lung injury. Pathological changes in lung tissues stained with H&E. Scale bar = 90 μm **(A)**, and the lung wet/dry (W/D) ratio **(B)** was detected in all groups. **p < 0.01 (by a one-way ANOVA). (*n* = 10 per group per experiment).

Next, we investigated the protective effects of the PEP-NASP in a mouse model of ALI induced by the i.n. administration of LPS. Lung histopathology was determined by H&E staining. We found that LPS administration resulted in obvious histopathological lung injury, including pulmonary edema, inflammatory cell infiltration, and alveolar damage, whereas transduction of the PEP-NASP markedly ameliorated these pathological changes (Figure 6A). There were no obvious pathological changes in the control group (Figure 6A). Edema is a typical pathological characteristic of ALI lungs. The elevated lung W/D weight indicated edema in LPS-challenged mice (Figure 6B). In contrast, the transduced PEP-NASP markedly decreased the W/D ratio compared to that in the ALI group (Figure 6B). These data suggested that the PEP-NASP alleviated the pulmonary pathological injury observed in LPS-treated mice.

### PEP-NASP Negatively Regulates LPS-Induced Production of Inflammatory Cytokines in the Lungs

**Figure 7:**
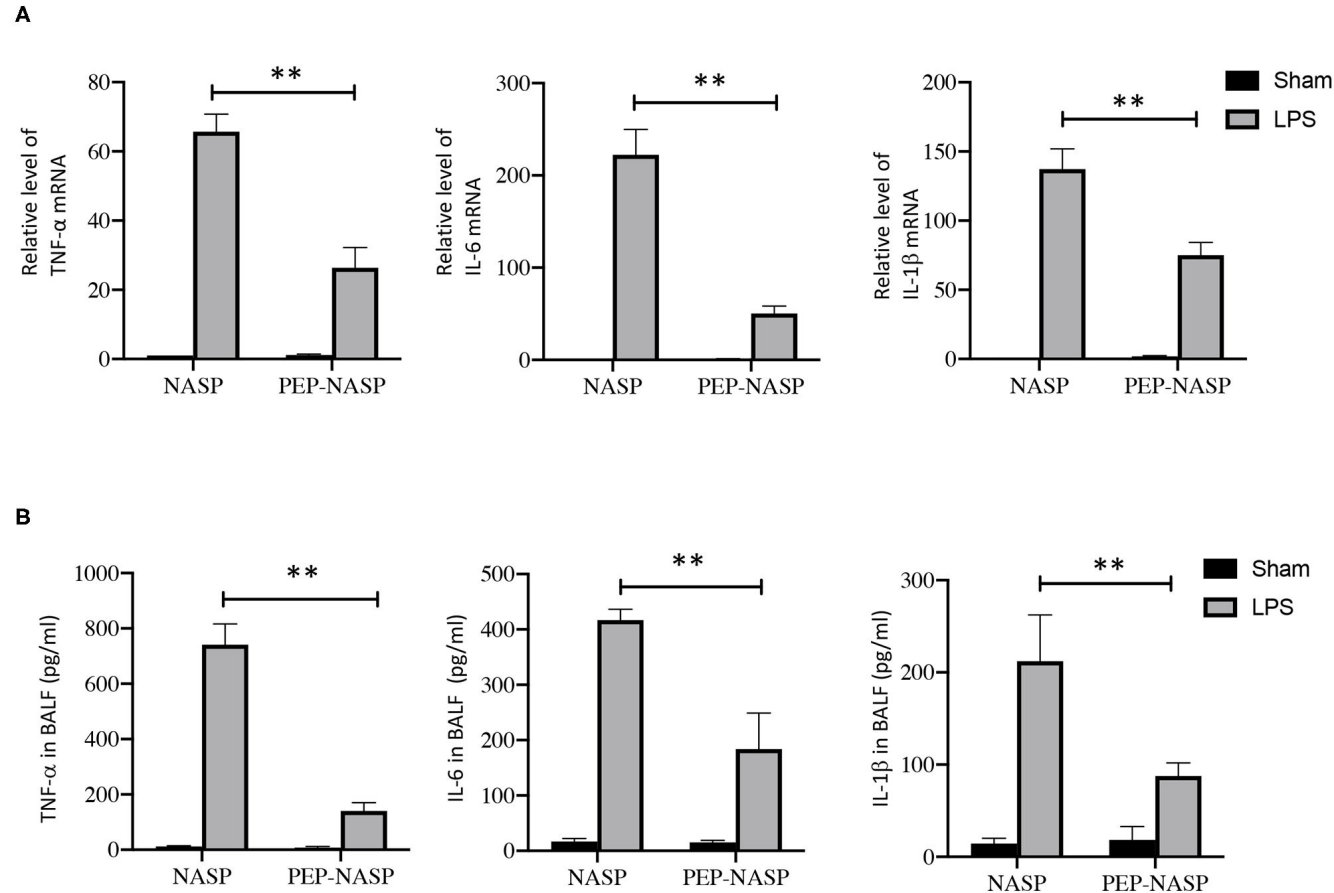
The PEP-NASP reduced expressions of TNF-α, IL-6, and IL-1β in mice treated with LPS. **(A)** RNA expressions of TNF-α, IL-6 and IL-1β in lung tissues were determined by an RT-qPCR. **(B)** Levels of proinflammatory factors (TNF-α, IL-6, and IL-1β) in bronchoalveolar lavage fluid (BALF) were detected by an ELISA. *N* = 10, **p < 0.01 (by a one-way ANOVA).

Expressions of inflammatory cytokines (TNF-α, IL-6, and IL-1β) in lung tissues were determined using a qPCR and ELISA. We found that mRNA expressions in lung tissues and protein expressions of TNF-α, IL-6, and IL-1β in BALF had dramatically increased in the LPS group (Figures 7A,B). By contrast, transduction of the PEP-NASP resulted in significant decreases in expressions of these cytokines (Figures 7A,B). These data indicated that the PEP-NASP negatively regulated expressions of inflammatory cytokines in LPS-induced lung injury.

### PEP-NASP Reduces Neutrophil Recruitment in the Lungs and Ameliorates Lung Tissue Damage During LPS-Induced ALI

**Figure 8:**
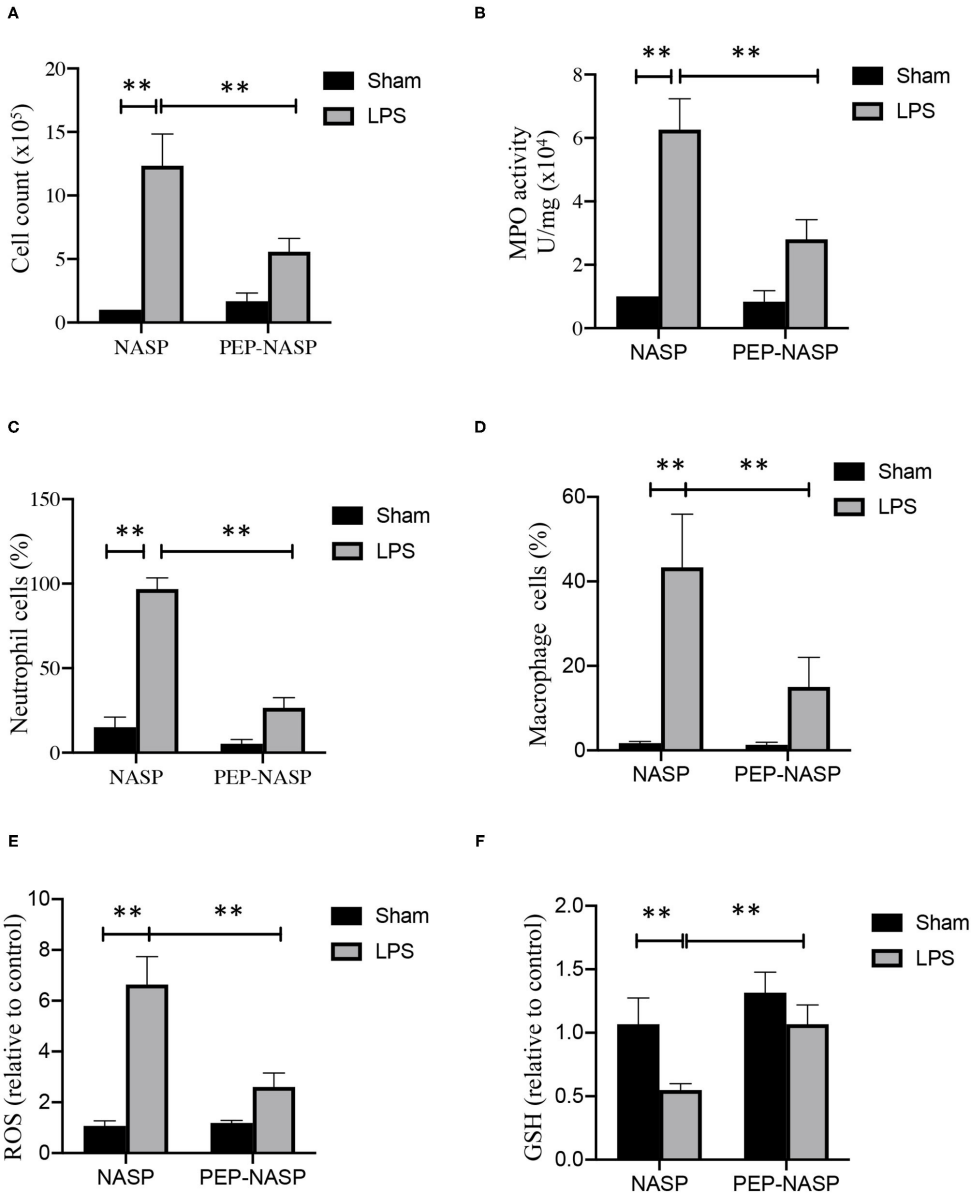
The PEP-NASP inhibited the infiltration of inflammatory cells in LPS-induced acute lung injury. **(A)** Total infiltrated cells in bronchoalveolar lavage fluid (BALF) were counted using a counting chamber. **(B)** Effect of the PEP-NASP on myeloperoxidase (MPO) activity in lung tissue homogenates. **(C,D)** Statistical analysis of percentages of neutrophils and macrophages in BALF by hemocytometer and Wright-Giemsa staining. **(E)** Production of ROS and **(F)** GSH level were measured in BALF. Values represent the mean ± standard error of the mean (SEM), *n* = 10, **p < 0.01 (by a one-way ANOVA).

Next, the infiltration of proinflammatory cells, including macrophages and neutrophils, was evaluated. Numbers of total cells in BALF had increased at 24 h after the LPS challenge, and numbers of total cells in mice with PEP-NASP pretreatment were significantly reduced (Figure 8A). Next, lung MPO activity was measured, as a specific marker for neutrophil infiltration and ALI severity, and LPS-challenged animals showed a significant increase in lung tissue MPO activity, while administration of the PEP-NASP dramatically reduced MPO activity (Figure 8B). We next explored the neutrophil and macrophage percentage changes in total cells by Wright-Giemsa staining. As shown in Figures 8C,D, percentages of neutrophils and macrophages had both significantly increased at 24 h after LPS challenge, while pretreatment with the PEP-NASP fusion protein reduced the percentage of neutrophils by 70% and macrophages by 28% compared to the LPS group. In addition, we also found that PEP-NASP fusion protein decreased the ROS production and increased the levels of GSH in mice of ALI (Figures 8E,F). These findings indicated that the transduced PEP-NASP suppressed ROS production and the infiltration of inflammatory cells into the lungs induced by LPS.

## Discussion

ALI, which is mainly characterized by pulmonary inflammation, is a common clinical disease in humans, and the mortality remains high at 30% ~ 40% in most studies (32, 33). Our previous study suggested that the sNASP had the ability to negatively regulate TLR signaling through targeting TRAF6 (16, 17). In a polymicrobial sepsis model, sNASP-mutant mice had defective bacterial clearance from the lungs due to marked reductions in IL-1β, TNF-α, and IFN-γ production associated with poor adhesion molecule expression and leukocyte recruitment, and defective phagolysosome formation. In this study, we demonstrated, for the first time, that administration of the PEP-NASP fusion protein effectively protected mice against LPS-induced ALI, with significantly attenuated pulmonary polymorphonuclear (PMN) infiltration, decreased lung MPO activity, suppressed inflammation, reduced BALF protein content, and ameliorated lung pathological changes. Moreover, we showed that the PEP-NASP alleviated the LPS-induced inflammatory response by inhibiting activation of the TLR/TRAF6 signaling pathway in macrophages *in vitro*. Thus, our data indicated that the PEP-NASP fusion protein conferred protection against lung injury induced by LPS.

Inflammation is a defense mechanism against exogenous pathogens, and it has been implicated in various aggressive and excessive inflammatory responses leading to serious tissue damage (34–36). Development of ALI is due to uncontrolled inflammation, which is connected to the overproduction of various inflammatory mediators released by diverse immune cells, such as endothelial cells, neutrophils, and macrophages. LPS, a major constituent of gram-negative bacterial cells, is a ligand of TLR4 that induces inflammatory syndromes, such as multiple organ injury and sepsis, and is generally recognized as a key factor in inducing ALI (35–37). Challenge of macrophages with LPS can produce high levels of NO, subsequently leading to proinflammatory and destructive effects mainly caused by iNOS (38, 39). In this study, we found that the PEP-NASP inhibited expressions of iNOS and COX-2 in LPS-stimulated macrophages. As is known, overproduction of inflammatory cytokines drives both COX-2 and iNOS gene expressions in macrophages, causing NO release (40). NO is a highly reactive free radical that participates in many inflammatory processes (41). Studies showed that *iNOS* and *COX-2* gene expressions markedly increased during ALI induced by LPS (42). These potential effects of the PEP-NASP on reducing NO suggest an anti-reactive oxygen species (ROS) role of the PEP-NASP in bacterium-induced inflammation.

TRAF6 is a key adaptor for TLR4/NF-κB signaling in regulating innate and adaptive immunity (43, 44). Increasing evidence indicates that the TLR4/TRAF6 pathway is considered to be involved in the process of inflammation-related ALI (45–47). For example, Ding et al. and Liu et al. both demonstrated that the inhibition of TRAF6 alleviated the severity of inflammation and lung injury in an ALI mouse model (45, 47). Inhibition of TRAF6 ubiquitination can attenuate TRAF6-mediated NF-κB activation and proinflammatory cytokine expressions (45, 46, 48). In recent years, many bioactive ingredients extracted from herbs have been reported to effectively ameliorate ALI *via* different mechanisms. For example, glycyrrhizic acid attenuates LPS-induced ALI *via* modulation of the PI3K/AKT/mTOR pathway (49); picrasma quassiodes attenuates LPS-induced pulmonary inflammation and pulmonary edema by regulating the iNOS, HO-1, NF-κB and MAPK signaling (50); and magnoflorine exerts anti-inflammatory effects against LPS-induced ALI through regulation of TLR4-mediated MAPK and NF-κB signaling pathways (51). Moreover, isoalantolactone, a traditional Chinese herbal medicine, was reported to improve pulmonary inflammation and permeability through inhibiting TRAF6 ubiquitination (45). Our findings indicated that the PEP-NASP prevented TRAF6 ubiquitination by directly interacting with TRAF6 in RAW 264.7 cells. Transduction of the PEP-NASP also negatively regulates TRAF6-downstream TAK1/p38/JNK and NF-κB activation. LPS-induced proinflammatory cytokines also decreased by the PEP-NASP in lung tissue *in vivo* and in macrophages *in vitro*. We also showed that the PEP-NASP inhibited activation of the NLRP3 inflammasome in RAW 264.7 cells. These results suggested that the PEP-NASP exerts its anti-inflammatory effects through suppressing TRAF6. Whether PEP-NASP regulate multiple levels of cellular process in response to LPS-induced inflammation is needed to be investigated in future studies.

Macrophages as important inflammatory cells were reported to attract neutrophil infiltration into the lungs and induce lung tissue damage (52). Meanwhile, clinical studies indicated that augmentation of the proinflammatory cytokines TNF-α, IL-1β, and IL-6 has a major role in the development of inflammatory diseases, including ALI (53). These cytokines amplify lung inflammation in ALI patients and were correlated with severity and poor outcomes, such as high protein pulmonary edema and respiratory failure (54, 55). The lung W/D ratio is an indicator of pulmonary edema. We found that LPS induced a higher lung W/D ratio than that of control mice, and administration of the PEP-NASP ameliorated the degree of pulmonary edema due to LPS. Higher levels of total proteins induced by LPS in BALF were also inhibited by the PEP-NASP. In addition, due to cytokines, neutrophils infiltrated into lung tissues, leading to the production of proinflammatory cytokines, which are secreted by activated macrophages in ALI patients and influence pulmonary gas exchange (56, 57). Inhibition of these proinflammatory cytokines provides important guidance for treating ALI. Our *in vivo* analysis performed in the ALI mouse model showed that the PEP-NASP suppressed the release of proinflammatory cytokines, including TNF-α, IL-1β, and IL-6, in lung tissues and BALF. These effects of the PEP-NASP on macrophages also revealed that the PEP-NASP might attenuate LPS-induced ALI *via* inhibiting cytokine production in macrophages. MPO is an enzyme stored in cytoplasmic granules of neutrophils, and its activity reflects the infiltration of neutrophils into lung tissues (58). We found that the PEP-NASP significantly decreased the activity of MPO induced by LPS and attenuated neutrophil infiltration into the lungs in LPS-induced ALI, indicating that the PEP-NASP can suppress neutrophil recruitment into lung tissues.

Studies have also found that the expression of TRAR6 was increased in lung tissues in mice with ALI (59–61). Clinically, TRAF6 variant (rs4755453) was significantly associated with susceptibility to sepsis-induced ALI in Chinese Han population (59). Therefore, it suggests that genetic variants in TRAF6 could influence TRAF6 mRNA stability and subsequently the production of inflammatory cytokines, which directly impacted lung tissue injury. The results of this study indicated the potent therapeutic effects of the novel peptide PEP-NASP against oxidative stress and pulmonary inflammation *via* selective TRAF6 signaling. Thus, PEP-NASP may exert potent therapeutic effects targeting genetic variants in TLR signaling, especially focused on high expression of TRAF6 population. We are planning to investigate the mechanism of PEP-NASP beyond genetic variants (rs4755453) in TRAF6 to further explore the clinical application.

In summary, we demonstrated for the first time that the human NASP, a negative inflammatory mediator, with its N-terminal fused to the PEP-1 peptide (PEP-NASP), can be efficiently transduced *in vivo* and *in vitro*. We also revealed that the transduced PEP-NASP has anti-inflammatory and antioxidant effects which ameliorated lung injury by regulating inflammatory responses and oxidative stress. The mechanisms for the protective role of the PEP-NASP are associated with the inhibition of TLR4-mediated TRAF6/NF-κB signaling pathways. Thus, our success in the protein transduction of PEP-NASP may provide a new therapeutic agent for protecting against inflammation-associated lung diseases, like ALI.

## Data Availability Statement

The raw data supporting the conclusions of this article will be made available by the authors, without undue reservation.

## Ethics Statement

The animal study was reviewed and approved by the Animal and Ethics Review Committee of the Laboratory Animal Center at Taipei Medical University.

## Author Contributions

F-MY, Y-CW, and S-PH developed the concepts. F-MY, Y-CW, and Y-TL designed and performed the experiments and analyzed the data. M-CH provided resource support. Y-CW contributed to the ALI animal model. M-CH, EY, Y-CW, and S-PH assisted in interpreting the data and writing, review, and editing. All authors contributed to the article and approved the submitted version.

## Funding

This work was financially supported of the Higher Education Sprout Project by the Ministry of Education (MOE) in Taiwan. F-MY was also supported by grants (TMU 108-AE1-B14, DP2-109-21121-01-T-04-02, and DP2-110-21121-01-T-04-02) from Taipei Medical University and (MOST 108-2320-B-038-069-MY2 and MOST 110-2320-B-038-065-MY3) from the Ministry of Science and Technology of Taiwan. Y-CW was also supported by grants (TMU 105-AE1-B53) from Taipei Medical University.

## Conflict of Interest

The authors declare that the research was conducted in the absence of any commercial or financial relationships that could be construed as a potential conflict of interest.

## Publisher's Note

All claims expressed in this article are solely those of the authors and do not necessarily represent those of their affiliated organizations, or those of the publisher, the editors and the reviewers. Any product that may be evaluated in this article, or claim that may be made by its manufacturer, is not guaranteed or endorsed by the publisher.
