# Supplementary figures and images for: PEP-sNASP Peptide Alleviates LPS-Induced Acute Lung Injury Through the TLR4/TRAF6 Axis
Source: Front Med (Lausanne). 2022 Mar 21;9:832713. doi: 10.3389/fmed.2022.832713 (PMC8977741; doi:10.3389/fmed.2022.832713)

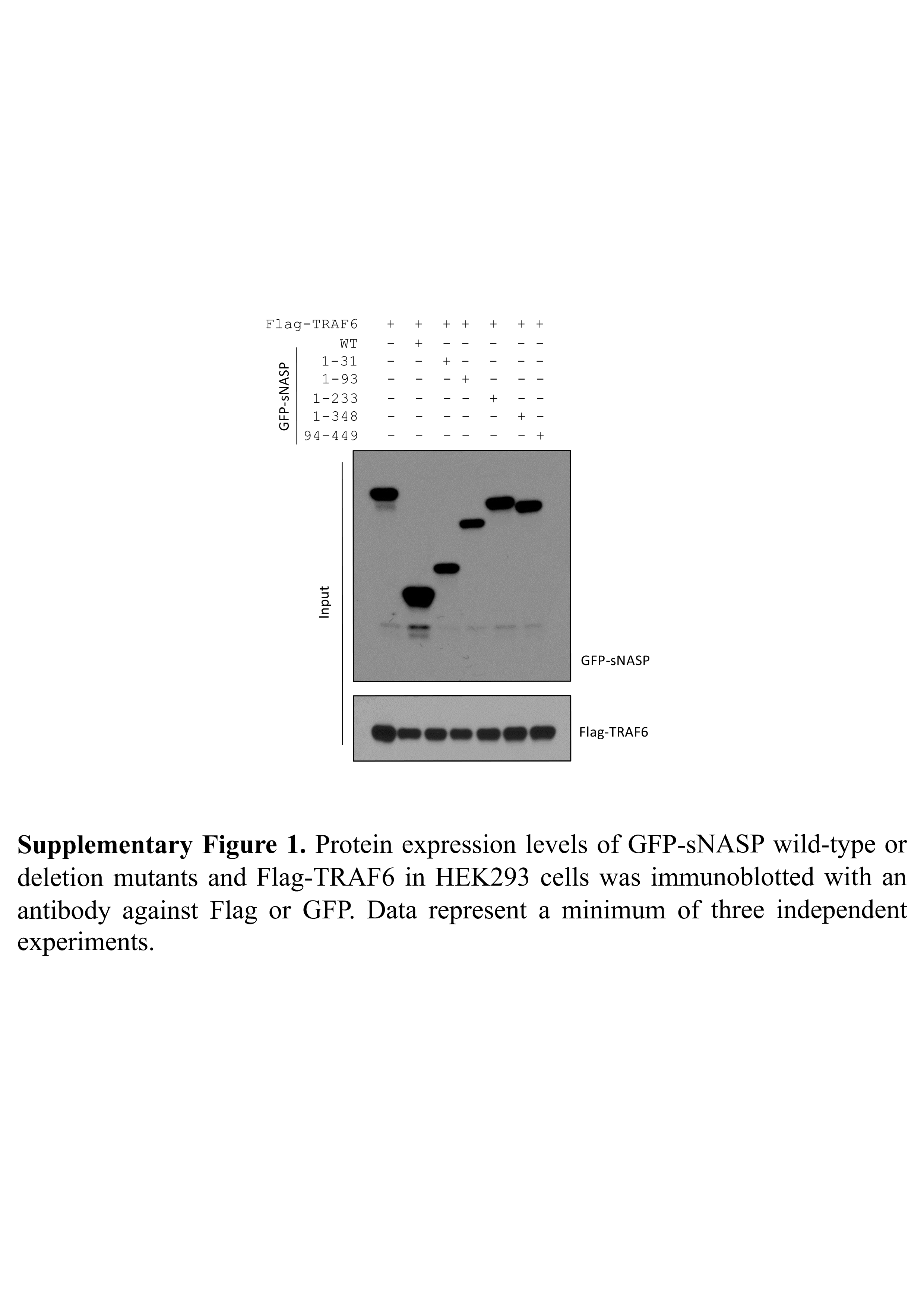

Supplement: Supplementary file 1 [file Image_1.TIF]

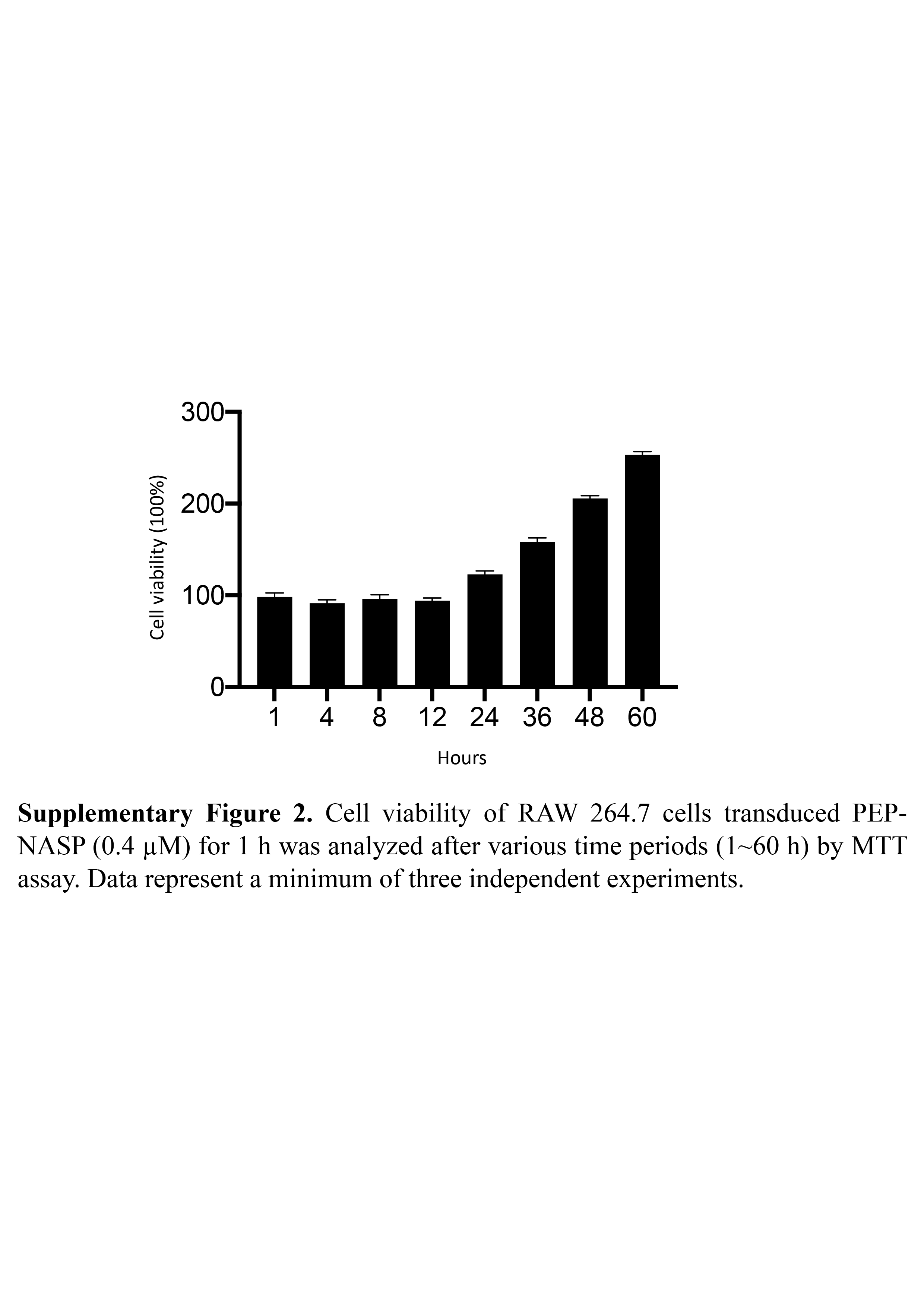

Supplement: Supplementary file 2 [file Image_2.TIF]
